# Recent Developments in Nanocomposite Membranes Based on Carbon Dots

**DOI:** 10.3390/polym16111481

**Published:** 2024-05-23

**Authors:** Shuheng He, Yiding Meng, Jiali Liu, Dali Huang, Yifang Mi, Rong Ma

**Affiliations:** 1 Key Laboratory of Advanced Textile Materials and Manufacturing Technology and Engineering Research Center for Eco-Dyeing & Finishing of Textiles, Ministry of Education, Zhejiang Sci-Tech University, Hangzhou 310018, China; 18815144900@163.com (S.H.); 15057142970@163.com (J.L.); 2 Artie McFerrin Department of Chemical Engineering, Texas A&M University, College Station, TX 77843, USA; 3 Zhejiang Institute of Standardization, Hangzhou 310007, China; mengyd@zis.org.cn; 4 Department of Materials Science & Engineering, Texas A&M University, College Station, TX 77843, USA; huangdali2010@gmail.com

**Keywords:** nanocomposite membrane, carbon dots, membrane separation, structure-performance

## Abstract

Carbon dots (CDs) have aroused colossal attention in the fabrication of nanocomposite membranes ascribed to their ultra-small size, good dispersibility, biocompatibility, excellent fluorescence, facile synthesis, and ease of functionalization. Their unique properties could significantly improve membrane performance, including permeance, selectivity, and antifouling ability. In this review, we summarized the recent development of CDs-based nanocomposite membranes in many application areas. Specifically, we paid attention to the structural regulation and functionalization of CDs-based nanocomposite membranes by CDs. Thus, a detailed discussion about the relationship between the CDs’ properties and microstructures and the separation performance of the prepared membranes was presented, highlighting the advantages of CDs in designing high-performance separation membranes. In addition, the excellent optical and electric properties of CDs enable the nanocomposite membranes with multiple functions, which was also presented in this review.

## 1. Introduction

Membrane technology is highlighted as a dominating separation technology ascribed to its high separation efficiency, versatility, cost-effectiveness, energy conservation, eco-friendliness, and safety [1,2]. It includes many different and distinctive separation processes, such as ultrafiltration (UF), nanofiltration (NF), forward osmosis (FO), reverse osmosis (RO), gas separation, pervaporation (PV), and ion exchange, providing a more holistic solution and making provision for substance purification, water treatments, as well as zero-carbon energy reconstruction [3,4,5]. However, there are still limitations that significantly impede the effectiveness of membranes in meeting the increasing separation standards and energy efficiency requirements. The first is the trade-off phenomenon between membrane permeability and selectivity, i.e., an increase in membrane permeability will result in a decrease in selectivity [6]. In addition, membrane fouling, including organic, inorganic, and biological fouling, is still an obstacle for the long-term applications of membranes because fouling can deteriorate membrane integrity and decrease the life span of membranes [7]. Therefore, sustaining improved membrane comprehensive performance, including permeability, selectivity, antifouling, and stability, is a strong incentive to trigger membrane design.

The development of nanomaterial technology opens an avenue for enhancing membrane performance. The combination of nanomaterials and polymer matrix realizes the simultaneously improved permeability and selectivity, successfully overcoming the trade-off effect [8,9]. Meanwhile, the functional nanomaterials can also endow the resultant membranes with additional features, such as antifouling, chloride resistance, antimicrobial capacity, photocatalytic ability, etc. [10]. Numerous nanomaterials, for instance, metal/metal oxides, graphene oxide (GO [11,12]), carbon nanotubes (CNT [13]), metal–organic frameworks (MOFs [8]), and covalent organic frameworks (COFs [14]), have been incorporated into polymeric membranes. Among them, carbon dots (CDs), as a rising star in the family of carbon-based nanomaterials, have aroused broad research interests for years due to their small size, good biocompatibility, abundant functional groups, and appreciable dispersibility. Since the first paper about CDs-based nanocomposite membranes was published in 2014, the articles related to CD nanocomposite membranes have increased dramatically. Benefits from the diverse properties of CDs, including particle size, hydrophilicity/hydrophobicity, as well as surface charges, can be applied in UF, NF, RO, PV, membrane distillation, etc. In this review, we summarized recent progress in CDs-based nanocomposite separation membranes. Specifically, we paid more attention to the structural regulation and functionalization of CDs-based nanocomposite membranes. Through analyzing the membrane formation process, the relationship between the CDs’ properties and microstructures, surface properties, as well as separation performance of the prepared membranes was constructed. Then, we discussed the reasons for the optical and electrical properties of CDs, and the functionalization of the nanocomposite membranes ascribed to the superiority of unique optical and electric properties of CDs in enhancing membrane performance were also presented in this review. These works highlight the advantages of CDs in designing high-performance separation membranes.

## 2. Carbon Dots

In 2004, Xu et al. occasionally found CDs when they segregated single-wall CNT and considered CDs as fluorescent nanoparticles at that time [15]. Until 2006, Sun et al. prepared carbon nanoparticles with various sizes and named them carbon quantum dots (CQDs [16]). Thenceforth, CDs have aroused colossal attention among researchers accounting for their unique properties. They possess ultra-small size, good dispersibility, biocompatibility, excellent fluorescence, facile synthesis, ease of functionalization, and chemical inertness [17,18]. Moreover, the tunable properties of CDs widen the scope of CDs even in applications limited to other carbon nanomaterials. Benefiting from the diverse functional groups on the surfaces, CDs are able to combine with organic and inorganic molecules via chemical treatments [19,20]. The electron-donor or electron-acceptor ability could be achieved by CDs depending on chemical structures, showing spectacular electronic properties [21]. These advantages suitably position CDs for applications in the fields of optoelectronics, catalysis, membrane separation, electrocatalysis, biomedicine, bioimaging, etc.

**Figure 1:**
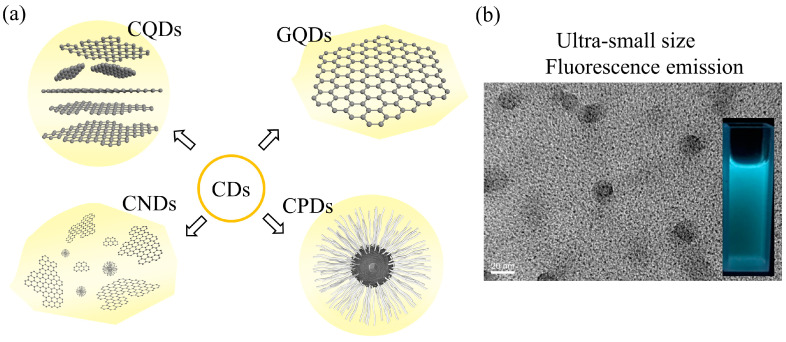
(**a**) Classifications of CDs: CQDs, GQDs, CNDs, and CPDs; (**b**) TEM and fluorescence emission optical image of CQDs.

Typically, graphene quantum dots (GQDs), CQDs, carbon nanodots (CNDs), and carbonized polymer dots (CPDs) are all adapted to be referred to as CDs (as shown in Figure 1) [22,23]. Therefore, it is necessary to define the structural features represented by these nouns. CQDs have a quasi-spherical structure, comprising a nanocrystalline core and amorphous regions on the surface. The core can be either crystalline or amorphous depending on the degree of sp^2^ carbon presented in the core. The surface of CQDs exhibits either hydrophilic or hydrophobic properties based on their functional groups. As a membrane of the graphene family, GQDs are 0D nanomaterials that have a 2D layered graphene core. They are anisotropic and lateral dimensions are typically smaller than 20 nm; CNDs are fluorescent carbon nanomaterials that lack quantum confinement, and their sizes are within 10 nm; CPDs consist of a polymer/carbon hybrid structure with a high number of carbon-based linkages on the surface and plenty of polymer networks in the interior, and their fluorescence is mainly enhanced by the crosslinking enhanced emissions effect. More detailed comparisons between GQDs, CQDs, CNDs, and CPDs are presented in Table 1.

### Advantages of CDs in Nanocomposite Membranes

CDs have aroused intense interest in the preparation of thin film nanocomposite (TFN) membranes ascribed to their unique characteristics. CDs are readily available nanomaterials and there are numerous approaches to obtaining CDs. CDs can be prepared through “top-down” and “bottom-up” methods. The top-down approach refers to breaking down large pieces of carbon materials via laser ablation, electrochemical oxidation, and arc discharge [22]. Meanwhile, CDs are also produced through the “bottom-up” method involves pyrolysis, hydrothermal, and microwave routes. Compared with the “top-down” method, the “bottom-up” method is a more facile and greener approach because no expensive precursors, sophisticated equipment, or complex procedures are needed. Moreover, it produces CDs from cheaper precursors, such as citrate, carbohydrates, proteins, and even other carbon-containing wastes [28]. The properties of CD can be facilely tuned by adjusting the compositions of precursors and preparation conditions. For instance, through doping with nitrogen, sulfur, and halogens, the characteristics of CDs including emission, photocatalytic capacity, and quantum yield can be enhanced [29]. In addition, CDs modified with amino groups can be involved in interfacial polymerization. The hydrophilicity of CDs is reduced with increasing pyrolysis time. The hydrophilic CDs are beneficial for water permeance and the antifouling ability of TFN membranes, while the hydrophobic CDs could accelerate non-polar solvent permeance. More importantly, CDs possess ultra-small size, good dispersibility, and low biotoxicity. The ultra-small size and good dispersibility of CDs avoid the aggregation of CDs in the polymer matrix and the functional groups on CDs’ surface such as hydroxy, carboxyl, amino, and sulfonic acid groups will improve the compatibility between CDs and polymer matrix through hydrogen bonding and electrostatic interactions. These merits make them suitable for constructing defect-free separation membranes.

## 3. Structural Regulation of CDs-Based Nanocomposite Membranes

Like other nanomaterials, CDs-based nanocomposite membranes can be prepared via interfacial polymerization, phase inversion, and surface engineering, which includes coating, layer-by-layer (LBL), assembly, and surface grafting. In this part, the addition of CDs on the microstructures and membrane performance was systematically analyzed, aiming to provide theoretical guidance for membrane structure optimization.

### 3.1. Thin Film Nanocomposite Membranes Prepared via Interfacial Polymerization

Interfacial polymerization has been widely applied to fabricate thin film composite (TFC) membranes [30,31]. This method utilizes polyamine monomers in the aqueous solution and acyl chloride monomers in the organic solution. The polyamine monomers are usually m-phenylenediamine (MPD), piperazine (PIP), and polyethyleneimine (PEI), while acyl chloride monomer is typically trimesoyl chloride (TMC). TFN membrane is a new type of composite membrane prepared via an interfacial polymerization process, which can break the trade-off relationship between permeability and selectivity by incorporation of nanomaterials within polyamide selective layers [32]. The origins of TFN membranes can be dated back to 2007 [33]. Hoek and his co-workers introduced zeolite NaA nanoparticles into the polyamide RO membrane, achieving a remarkable water flux enhancement without sacrificing salt rejections. The super hydrophilic and molecular sieve nanoparticle pores created water transport channels within the polymeric matrix, where water molecules tend to flow preferentially. Since then, numerous nanomaterials have been adopted to fabricate TFN membranes. Meanwhile, the incorporated nanomaterials need to meet some criteria: (1) considering the thickness of membranes, the particle size of nanomaterials should be within 200 nm; (2) these nanomaterials should have good dispersibility and compatibility with polymer matrix to avoid the generation of non-selective nanovoids in the selective layers [34]; and (3) the employed nanofillers should be stable and as inexpensive as possible. CDs possess many characteristics such as small particle size (within 20 nm), good water dispersibility, and ease of modification. These merits make them ideal nanofillers to construct TFN membranes.

CDs are usually added to the aqueous solution of amine monomers to fabricate TFN membranes. Ascribed to remarkable water solubility, CDs can uniformly disperse in aqueous phase alongside amine monomers. With the incorporation of CDs, the resultant membranes demonstrate enhanced permeability, solute selectivity, and other properties such as antifouling and chlorine tolerance. Moreover, the performance can be further improved by proper optimization of CDs’ characteristics. Therefore, the CDs-incorporated TFN membranes have been widely applied in NF, organic solvent NF (OSN), RO, PV, FO, and gas separation processes [35,36].

**Figure 2:**
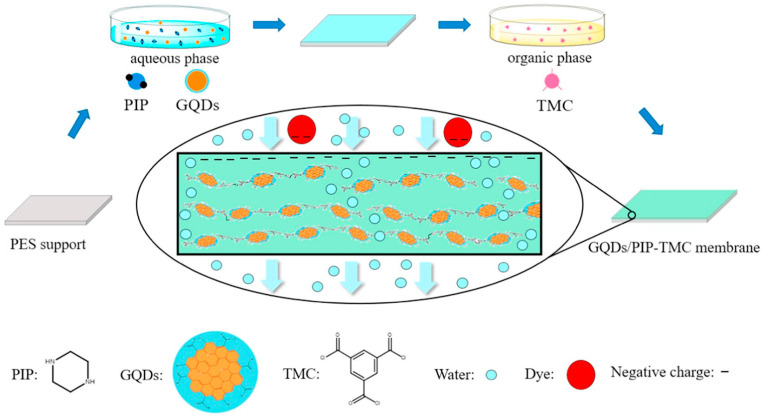
Preparation of CDs-based TFN membranes during interfacial polymerization [37].

Bi et al. [37] presented high water flux and antifouling TFN membrane by the incorporation of GQDs into polyamide NF membranes (Figure 2). The separation layers with extra water channels created by GQDs were thinner, smoother, and more hydrophilic compared with pristine polyamide layers, facilitating water transport and weakening the non-specific adsorption of foulants. Consequently, the water flux of the GQDs-incorporated TFN membrane was enhanced about four times even under hash fouling conditions. The abundant functional groups on CDs’ surfaces ensure great opportunities to achieve a fit-for-purpose design of TFN membranes via further modifications, compared to other inorganic nanomaterials [38]. Xu et al. [39] presented a novel TFN NF membrane with the addition of amine-modified GQDs (GQDs-NH_2_). GQDs-NH_2_ combines the inherent advantage of GQDs with the chemical properties of amine groups, effectively regulating the structures and properties of NF membranes. The amine groups on GQDs-NH_2_ lead to a more positively charged membrane surface, and thus, the resultant NF membrane possessed excellent Mg^2+^/Li^+^ separation capability. The mass ratio of Mg^2+^/Li^+^ decreased prominently from 20 to 0.7. In addition, the water flux was 137.8% more than that of the pristine membrane ascribed to the water channel created by GQDs-NH_2_ and hydrophilic surface. Furthermore, Sun et al. [40] systematically analyzed the effect of ionic groups on CQDs on the prepared TFN NF membrane separation performance. Ascribed to the superiorities of CQDs’ remarkable hydrophilicity and ultra-small size, the TFN membranes revealed higher permeances along with well-maintained rejections of sodium sulfate (Na_2_SO_4_), as well as improved antifouling capacities in comparison with TFC membranes. In particular, the sulfonic acid groups modified CQDs contributed to a looser and more negative polyamide selective layer, endowing the corresponding TFN NF membrane with the highest water permeability of 7.01 L m^−2^ h^−1^ bar^−1^ and Na_2_SO_4_ rejection of 93.6%. The amine-modified CQDs (NCQDs) showed better adhesion strength with the polyamide matrix and their positive charge resulted in a less negative surface charge of membranes; consequently, the TFN NF membrane incorporated NCQDs exhibited better retention of divalent cations. Gai et al. [41] found that the state of carboxyl groups on CQDs also influences the performance of TFN membranes. CQDs were obtained by pyrolysis of citric acid and Na^+^-CQDs were prepared by adjusting the CQDs solution to a pH of 8 with a sodium hydroxide solution. They observed that the addition of CQDs resulted in a thinner and smoother selective layer due to the acidic properties of CQDs. On the contrary, Na^+^-CQDs contributed to thinner and rougher membranes because Na^+^-CQDs induced additional interstitial space for MPD diffusing towards the organic phase and reacting with TMC. Compared with pristine TFC membrane, the TFN-CQDs exhibited lower water flux and NaCl rejections, while the water flux of TFN-Na^+^-CQDs dramatically boosted to 34.86 L m^−2^ h^−1^ bar^−1^ without sacrificing NaCl rejection.

We summarized the characteristics and performances of CDs-based TFN membranes in Table 2. The incorporation of CDs mostly generates thinner and smoother selective layers compared to pristine membranes; however, some studies also reported rougher TFN membranes. It is known that the diffusion behavior of amine monomers not only decides the reaction rate but also determines the final microstructures of selective layers [42]. CDs are larger than amine monomers in size and therefore sterically hinder the diffusion of amine monomers. In addition, CDs can interact with amine monomers through electrostatic interactions, hydrogen bonding, and/or π-π interaction. As a result, the diffusion of amine monomers could be regulated by the incorporated CDs, effectively tuning the microstructures of selective layers. More importantly, the interactions between amine monomers and CDs are diverse due to the characteristics of amine monomers and CDs, providing a broader possibility for membrane structural regulation.

**Figure 3:**
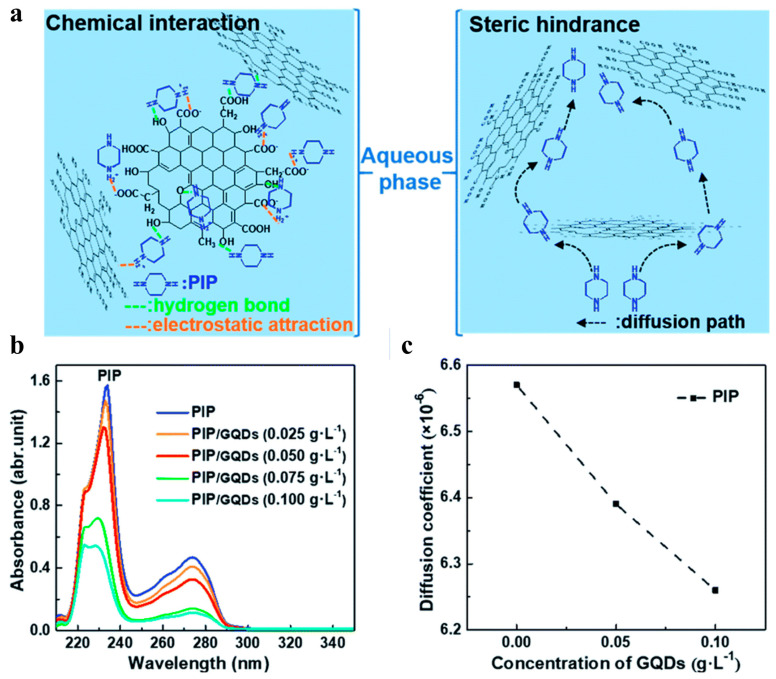
(**a**) The possible interactions between GQDs and PIP. (**b**) UV-vis spectra of PIP in the organic phase with various GQDs concentrations. (**c**) The influence of GQDs concentration on diffusion coefficients of PIP [56].

**Figure 4:**
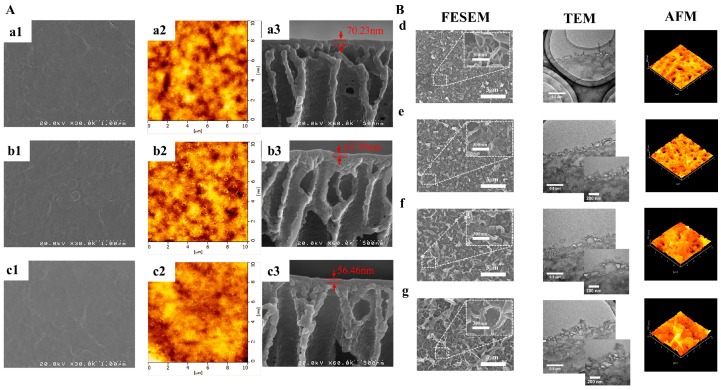
The scanning electron microscope images and transmission electron microscope images of membranes. (**A**) Thinner and smoother TFN membranes after incorporation of GQDs. The concentrations of GQDs were: (**a1**–**a3**) 0 wt%, (**b1**–**b3**) 0.03 wt%, (**c1**–**c3**) 0.1 wt% [39]; (**B**) rougher and thicker TFN membranes after incorporation of GQDs. (**d**) polyamide NF membrane, (**e**) GOQD-polyamide NF membrane, (**f**) N-GOQD-polyamide NF membrane, (**g**) N/S-GOQD-polyamide NF membrane [64].

The thinner and smoother membrane surface is ascribed to the low diffusion rate of PIP caused by the incorporated CDs (Figure 3). The electrostatic repulsion or hydrogen bonding between cationic amine monomers and cationic CDs arouses greater resistance for amine monomers to diffuse deep into the organic phase [39,45,61]. Meanwhile, the diffusion of PIP is also affected by the steric hindrance of CDs. Both reasons will result in thinner and smoother TFN selective layers [37,48] (Figure 4A). The rougher surface (as shown in Figure 4B) may be caused by the following: (1) The anionic groups on CQDs enable the adsorption of amine monomers on substrate membranes, which extends the reaction region of two immiscible phases and thus leads to the formation of rougher membrane surface [40]. (2) CDs with amino groups would be more prone to react with TMC to form initial amide cluster structures, resulting in rougher surfaces. CDs impede the polymerization reactions between the two-phase monomers and create more diffusion channels for amine monomers in the initial polyamide network. Consequently, more amine molecules could diffuse across the initial polyamide network to react with TMC molecules, leading to a denser, rougher, and thicker membrane [61]. (3) The size of CDs will influence the membrane roughness. Zheng et al. found that a larger zwitterionic shell of CDs accumulated more PIP around the CDs, which resulted in intense reactions with TMC molecules forming sparsely distributed bulges on the membrane surface. This molecular attraction is more dominant than the steric hindrance effect with the increase in the zwitterionic shell size of CDs; consequently, a rougher surface membrane is observed [50].

**Figure 5:**
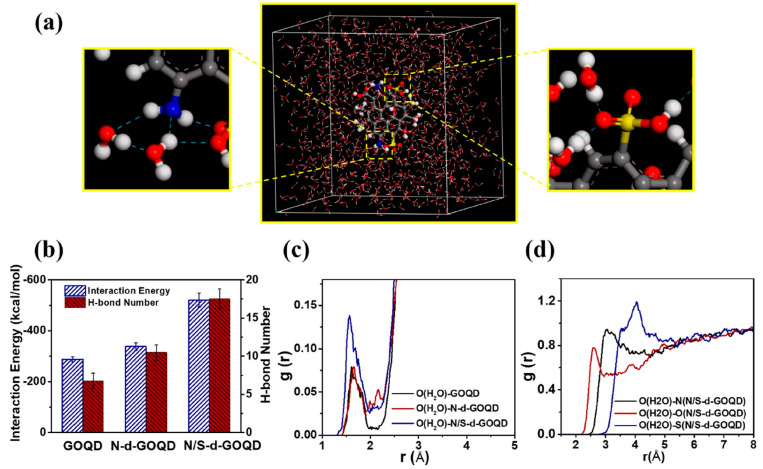
(**a**) Molecular model of N/S-d-GOQD (gray—C atom, white—H atom, blue—N atom, red—O atom) in water and dotted blue line indicated interactions between the GQDs with water molecules; (**b**) interaction energy and intramolecular H-bonds number between GOQD, N-d-GOQD, and N/S-d-GOQD with water molecules; (**c**) RDF analysis of water centroid of GOQD, N-d-GOQD, or N/S-d-GOQD interactions, respectively; and (**d**) RDF analysis of oxygen atom (O(H2O)) of water with the oxygen atom of carboxylic, ammonia, and sulfonic groups [64].

The significant increase in water permeation can be interpreted as follows: (1) interfacial voids created between CDs and polymeric matrix lower the intrinsic resistance of membranes and serve as water transport channels for fast permeation; (2) the thinner selective layer will be beneficial for water permeation or the rough surface contributes to the enlarged surface area for water permeation; and (3) the functional groups will enhance internal polarity with improved surface hydrophilicity. Shen et al. [64] implemented MD simulation to disclose the underlying interactions between water molecules and GQDs with different functional groups (Figure 5). Compared to GQDs and GQDs-NH_2_, sulfonic-acid-modified GQDs showed much stronger interaction energy with water molecules. This indicated that sulfonic-acid-modified GQDs owned better water capture ability ascribed to the stronger hydrogen bond interactions with water molecules.

**Figure 6:**
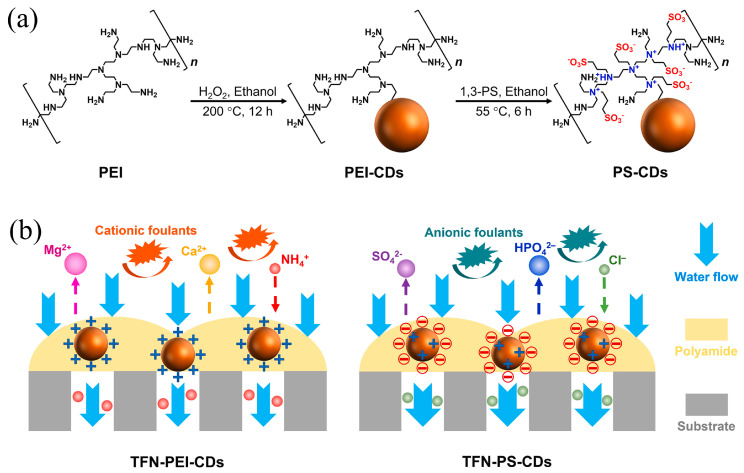
(**a**,**b**) Schematic of separation behavior of TFN membranes influenced by CDs with different functional groups [48].

Moreover, it is found that the functional groups on CDs not only tune the microstructures of the selective layer but also tailor the solute rejections of the resultant TFN membranes. Normally, the incorporation of CDs with hydroxyl, carboxyl acid groups, and sulfonic acid would bring about more negatively charged membrane surfaces, while CDs with amine groups would enhance the surfaces’ positive charge. As shown in Figure 6, when these CDs are incorporated into polymeric membranes, the formed charged nanovoids could strengthen dielectric exclusion effects, together with the Donnan effect, and endow the TFN membranes with high rejections of divalent co-ions [40,48,61]. Therefore, the TFN membrane successfully overcomes the trade-off between water permeability and solute selectivity.

The merits of CDs also have a remarkable influence on membranes’ hydrophilic–hydrophobic properties, which in turn affects their application performances. Mazhari et al. [62] incorporated biowaste-derived nitrogen-doped CQDs (BWD-NCQDs) into the TFC selective layer. The improved membrane hydrophilicity in addition to the generated nanovoid, both were beneficial for the improved FO proficiency in water desalination. During the FO process, the water flux increased by 54.7% while the reverse solute flux decreased by 43.3%. Apart from the hydrophilicity of CDs, the hydrophobicity of CDs also contributes to enhancing TFN membrane performance. Yuan et al. [72] tailored the carbonation degree of CDs to govern the permeation of different organic solvents. CDs with a low degree of carbonization possessed more hydrophilic groups and could efficiently adsorb polar solvents, thus providing a 54.3% permeance enhancement for isopropanol to 4.26 L m^−2^ h^−1^ bar^−1^. On the contrary, the non-polar solvent permeation was obviously improved by the incorporation of highly carbonized CDs via their hydrophobic domains.

**Figure 7:**
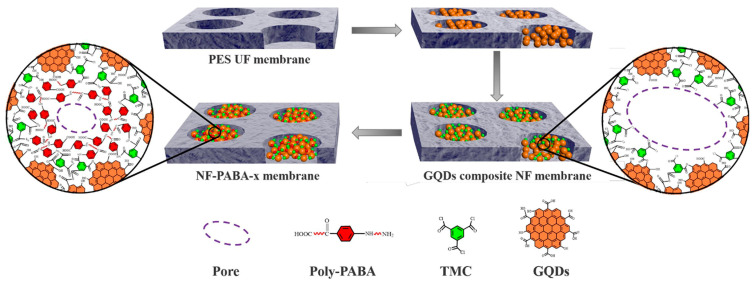
Schematic of pore size regulation by GQDs and PABA [74].

Attributed to their ultra-small size and glorious dispersity, CDs can also be selected to engineer the pores of UF membranes. If the diffusion of CDs is slow, the interfacial polymerization eventually occurs within the pores of UF membranes. Through controlling the GQDs amount, TFN NF membranes with tunable nanochannels were constructed by Jiang’s groups [73]. The radius of the resultant TFN membranes could range from 1.21 to 1.72 nm, realizing ultrafast water permeation of 244.7 L m^−2^ h^−1^ bar^−1^ and high rejection to Alcian blue (92.9%) and Congo red (98.8%). Then, they [74] used *p*-aminobenzoic acid to further modify the GQD composite NF membrane (Figure 7). The obtained membranes exhibited a high water permeance of 128.1 L m^−2^ h^−1^ bar^−1^ and selectivity factors for Alcian Blue/Na_2_SO_4_ and Evans Blue/Na_2_SO_4_ were as high as 877.0 and 175.4, respectively.

**Figure 8:**
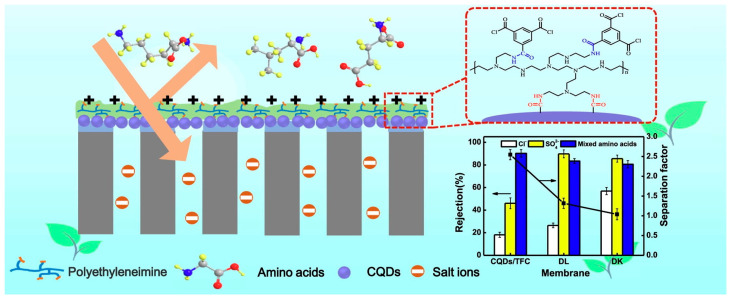
Separation mechanism of CQDs/polyamide membrane in amino acid desalination [76].

Furthermore, CDs can be pre-incorporated on the substrate as an interlayer to control the interfacial polymerization process. This hydrophilic interlayer effectively regulates the diffusion of amine via hydrogen bonding and electrostatic interactions, resulting in thinner and smoother selective layers [75]. The thinner and intact selective layers are crucial for maintaining high permeation without decreasing solute selectivity. For example, Yang et al. [76] conducted interfacial polymerization between PEI and TMC on a CQDs-modified polyethersulfone (PES) substrate (Figure 8). The carboxyl groups of CQDs on the substrate were involved in the reaction with PEI molecules, leading to a looser upper selective layer. The water permeability enhanced significantly from 3.6 to 9.7 L m^−2^ h^−1^ bar^−1^ and rejections towards amino acids were all above 90%. In addition, Liang et al. [77] modified polyimide substrates via depositing hydrophilic GQDs with the aid of PEI. This interlayer covalent bonded with the substrate and upper selective layers, contributing to membrane stability in organic solvent filtration. With the aid of GQDs interlayer, the thickness and roughness of the OSN membrane decreased remarkably. As a result, this GQDs-interlayered OSN membrane demonstrated increased ethanol permeance (4.03 L m^−2^ h^−1^ bar^−1^) and Rhodamine B rejection (98.7%). Niu et al. [67] used the amino-functionalized GQDs (NGQDs) as an interlayer to modify the PES UF substrate, where the interfacial polymerization between β-cyclodextrin and TMC was conducted. The constructed loose interlayer provided effective transport channels for CO_2_ diffusion and the affinity of amino and amide groups attributed to NGQDs and polyamide contributed to CO_2_/N_2_ selective separation. Thus, the CO_2_/N_2_ selectivity was as high as 23.3 with a CO_2_ permeance of 174.5 GPU.

### 3.2. Phase Inversion Nanocomposite Membranes

**Figure 9:**
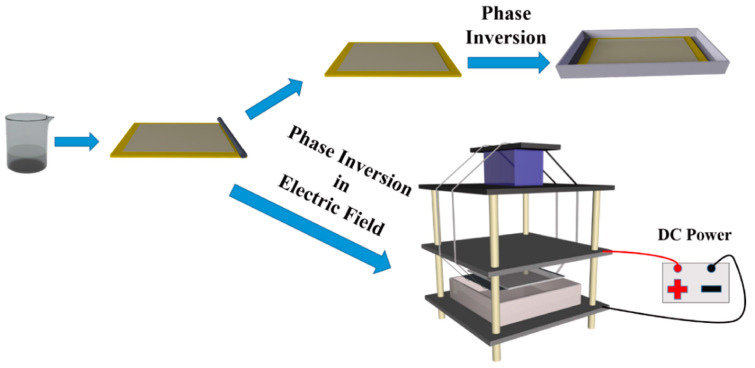
Preparation of PES/CQDs membrane via phase inversion with/without DC electric field [84].

Phase inversion is also commonly used to prepare mixed-matrix membranes. In a typical procedure of phase inversion process, a casting solution containing CDs and dissolved polymers is bladed on a substrate, and then immersed in a coagulant bath for phase separation by solvent exchange, forming an asymmetric membrane with a thin, dense skin layer and porous bottom layer [78]. It is facile to fabricate tunable nanocomposite membranes via phase inversion as the presence of CDs in the casting solution influences the thermodynamics and kinetic factors, strongly changing the morphology and pore structure of membranes [79]. The hydrophilic CDs can strengthen the mass transfer speed between the solvent and non-solvent, creating large membrane pores and channels. In addition, CDs migrated on the membrane surfaces increase membrane hydrophilicity, which is beneficial for enhancing antifouling ability. Koulivand et al. [80] blended CDs into a PES matrix. The permeability raised to 76.5 kg m^−2^ h^−1^, which was almost twice the bare PES, with dye rejections all above 97%. Meanwhile, the irreversible flux decline ratio decreased from 46.3% to 20.3%. Similarly, Yang et al. [81] incorporated CQDs, tertiary amine CQDs (TQDs), and zwitterionic CQDs (ZQDs), respectively, into cellulose acetate–polyurethane-based tubular membranes for efficient removal of copper ions. The functional groups of CQDs on the membrane morphologies and membrane performance were fully analyzed. TQDs possess hydrophilic amine groups compared to CQDs, leading to TQD membranes being more porous than CQDs membranes; ZQDs intensively interact with water molecules via ionic solvation, generating the most porous membrane (ZQDs membrane, ZQDs-M). Hence, the ZQDs membrane displayed a high water permeance of 6277.4 L m^−2^ h^−1^ bar^−1^ with a 95.4% rejection of copper ions. Meanwhile, ZQDs-M achieved prominent anti-adhesion and antifouling properties. In addition, Vatanpour et al. [82] found that the membrane with 2 wt% GQDs possessed the smallest pore size and lowest porosity compared to the membrane with 1 wt% GQDs. This is because the viscosity will be enhanced by the addition of CDs, delaying the phase separation process and preventing instant demixing. Under this situation, even though the hydrophilic CDs accelerate the mass transfer rate, the influence of viscosity dominates the phase inversion process and reduces the porosity and pore size of the membrane [83]. To solve this issue, Zhang et al. [84] applied a direct current (DC) electric field during the PES/CQDs phase inversion process (Figure 9). The PES molecular chains are polarized under a DC electric field and their conformation changes from topological entanglement to cohesive entanglement. The ordered array of polarized PES molecular chains can avoid the increase in viscosity of the casting solution caused by CQDs, further promoting the exchange rate of the solvent and water. Moreover, hydrophilic CQDs can modify some poorly hydrophilic nanoparticles such as UiO-66-NH_2_ and silver nanoparticles to enhance their dispersion, and the synergistic effects of CQDs and these nanoparticles provide a new avenue for the fabrication of high-performance phase inversion membranes [85,86].

### 3.3. Surface Engineering Nanocomposite Membranes

CDs-based nanocomposite membranes can be fabricated using other methods, encompassing coating, LBL, assembly, and surface grafting.

In the coating method, mixture solutions containing CDs and polymers are cast on the substrate followed by solidification in a drying step. Parthiban et al. [87] explored the CQDs as potential nanofillers to realize a Nafion hybrid membrane via solution coating. The hydrophilic groups on CQDs were hydrogen bonded with sulfonic acid groups in the Nafion matrix, promoting ionic conductivity. Compared to the pristine Nafion membrane, the nanocomposite membrane realized about a 33% decline in methanol crossover and about 30% higher proton conductivity. Jin et al. [88] studied the influence of CQDs’ functional groups on ionic conductivity. Compared to GQDs and oxygen-containing CQDs (O-CQDs), nitrogen-containing CQDs (NO-CQDs) achieved the highest interaction energy with water molecules, indicating the best water capture ability. The superior water capture ability of NO-CQDs promoted a well-defined microphase-separated structure and accelerated the transport of hydroxide ions through the formation and breaking of hydrogen bonds. Furthermore, CDs can act as carriers to incorporate other functional components and ensure their stability within the membrane matrix. For example, Huang et al. [89] designed a novel proton exchange membrane (PEM) containing hindered amine-grafted CQDs to improve the chemical stability of PEM by eliminating active free radicals. CQDs could form physical crosslinking sites with perfluorosulfonic acid chains through hydrogen bonds, ensuring themselves stable in nanocomposite PEM. Thus, the loss of hindered amine was avoided by designing hindered amine-grafted CQDs, endowing the resultant PEM with stable free radical elimination ability.

**Figure 10:**
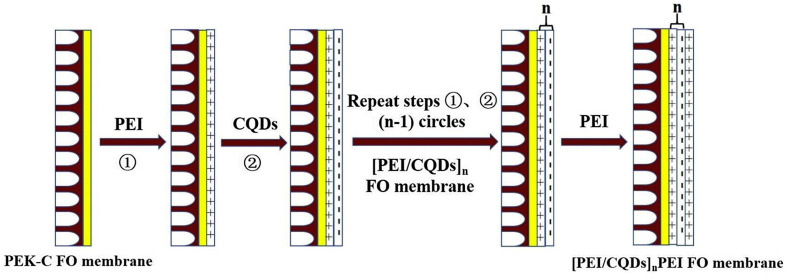
Preparation of [PEI/CQDs]_n_ FO membranes [91].

LBL is a versatile method to produce nanoscale thin membranes by adsorbing oppositely charged polyelectrolytes or nanoparticles sequentially onto a substrate through various interactions [90]. CDs with diverse functional groups can be either positively charged or negatively charged; therefore, CDs can interact with oppositely charged polyelectrolytes. For instance, Deng et al. [91] prepared FO membranes via LBL assembly by using positive PEI and negative CQDs (Figure 10). The thickness, surface charge, and performance of the resultant membranes can be optimized by assembly cycles, the polyelectrolyte or CD solution concentrations, the ionic strength of polyelectrolytes, and the adsorption time. When the assembly number was 2, [PEI/CQDs]_2_PEI exhibited the best water flux of 30.8 L m^−2^ h^−1^ and a reverse salt flux of 8.9 g m^−2^ h^−1^, respectively, surpassing most of the reported FO membranes.

**Figure 11:**
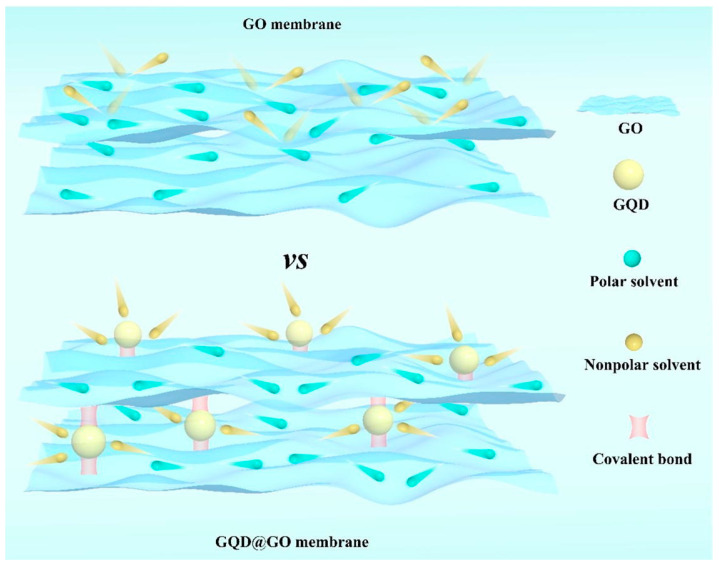
The adjustment of nanochannels of GO membranes by GQDs [95].

Two-dimensional lamellar membranes, featuring unique mass transport and exceptional selectivity, open an avenue for membrane technology [92]. The separation characteristics mainly rely on horizontal interlaminar nanochannels. The microenvironment of these interlaminar nanochannels, including the proper and stable channel size and suitable chemical affinity, is crucial for ensuring stable permeance and selectivity [93,94]. Wu et al. [95] introduced hydrophobic GQDs to manipulate the microenvironments of GO nanochannels to permit fast nonpolar molecule transport (Figure 11). The hydrophobic regions created by GQDs facilitated the dissolution of nonpolar molecules and enlarged nanochannel size (1.35 nm) ensured their rapid fast transport. The synergistic effect collectively enhanced the permeance of acetone and h-hexane to 173.5 and 85.4 L m^−2^ h^−1^ bar^−1^, respectively. Meanwhile, the membrane exhibited structural stability under acidic and alkaline conditions ascribed to covalent bonds formed between GQDs and GO. Compared with other intercalators, GQDs could be better distributed on GO nanosheets ascribed to their compositional similarity [96]. Similarly, Zhang et al. [97] chose hydrophilic CQDs as rigid nano-wedges to enlarge the interlaminar nanochannels for efficient n-butanol dehydration. The hydrophilic and enlarged nanochannels endowed the membrane with a remarkable permeation flux of 8877.8 g m^−2^ h^−1^ while the separation factor can reach 3763. Moreover, the functional groups on CDs can also change the chemical environment of interlaminar nanochannels. For instance, positively charged CQDs introduced positive charge within negatively charged GO membranes; as a result, the membrane can effectively reject negatively, neutral, and positively dissociative personal care products [98]. In addition, CDs can influence the growth of some lamellar nanomaterials, and the in situ formation of nanocomposite membranes. Liu et al. [99] used GQDs to inhibit the growth of molybdenum disulfide (MoS_2_) nanosheet. The obtained MoS_2_/GQDs membrane achieved improved water permeance (64.5 L m^−2^ h^−1^ bar^−1^) and methanol permeance (78 L m^−2^ h^−1^ bar^−1^) with high Evans blue rejection.

Membrane surface properties have a major impact on the membrane performance in terms of separation and antifouling characteristics. CDs have the advantages of tunable hydrophilicity/hydrophobicity and various functional groups, making themselves ideal candidates for modifying membrane surface properties. The hydrophilic GQDs can be physically bound on polyamide NF membranes through pressure-assisted filtration [100]. The improved membrane hydrophilicity and electronegativity contributed to increased water permeance and salt/dye rejections, respectively. Even though GQDs were physically bound, the negligible detachment of GODs was evidenced during the long-time stability test. Benefiting from amine or carboxylic acid groups, hydrophilic CDs are able to chemically bond on membrane surfaces, endowing the membrane with highly bactericidal [101], antibiofouling [102,103,104], and efficient acid/chlorine resistance [105]. In addition, the hydrophobic CDs provide the possibility for the construction of oil–water separation membranes through surface modification. Lei et al. [106] fabricated a hydrophobic membrane simply by surface functionalization of cotton fabrics via octadecylamine-functionalized CQDs. The transition from hydrophilic to hydrophobic surface made the membrane achieve efficient oil−saltwater separation (up to 99%), and this modification did not sacrifice the highly permeable water vapor of cotton fabrics, which is suitable for water desalination.

## 4. Photocatalytic/Photoluminescent/Conductive Functionalized CDs Based Nanocomposite Membranes

The potential of CDs in fabricating high-performance membranes has been fully explored due to their ultra-small particle size, facile synthesis, easy surface modification, good water dispersibility, biocompatibility, and chemical inertness. Except for the above-stated merits, CDs also own outstanding photoluminescence, photochemical, and conductive activity. This part summarizes the applications of optical and electronic properties of CDs in improving nanocomposite membranes’ performance.

Membrane fouling reduces separation efficiency and shortens the lifetime of membranes, which is an obstacle to the practical applications of membranes. Photocatalytic membranes provide a viable solution to mitigate membrane fouling. It integrates membrane separation and photocatalytic functions in one operating module, which can not only degrade the foulants on the membrane surface but also remove and degrade harmful substances in water. The design of photocatalytic membranes has been a hotspot in membrane research.

Efficient photocatalysts are the core of designing photocatalytic membranes. The limited light utilization and high recombination rate of photoinduced electrons and holes both seriously restrict the efficiency of photocatalysts. CDs unfold prominently optical and electronic properties, including light absorption, photoluminescence (PL), up-converted photoluminescence (UCPL), and photoinduced electron transfer [107]. The light absorption of CDs is related to their surface state, which can be varied with the diverse precursor and preparation methods. The fluorescence emission wavelength of CDs can be shorter than their excitation wavelength attributed to the brilliant UCPL property. Both features render CDs suitable for the fit-for-purpose design of CDs-based photocatalysts by full utilization of solar light. In addition, the PL of CDs can be quenched by either electron donor or electron acceptor molecules, demonstrating the remarkable electron transfer ability. Thus, attributed to unique PL behavior, UCPL emission, as well as photoinduced electron transfer properties, CDs themselves can be used as a photocatalyst, as a photosensitizer to improve the light absorption ability of other catalysts, and as an electron mediator to inhibit the recombination of photogenerated electrons and holes. To sum up, CDs achieve huge potential in the field of photocatalysis.

Shao et al. [108] constructed a dual functional CQDs interlayer between the PES substrate and polyamide layer, offering a self-cleaning TFN NF membrane. On the one hand, the CQDs interlayer reduced the solute transport resistance and alleviated concentration polarization; on the other hand, these photoactive CQDs could degrade organic dyes deposited on the membrane surface under visible light irradiation. In addition, the photocatalytic properties of CQDs could be significantly influenced by functional groups [49,109]. Yu et al. [49] modified GQDs with five kinds of amino acids and prepared self-cleaning NF membranes via interfacial polymerization. The results showed that L-cysteine functionalized GQDs possessed the narrowest bandgap energy and improved the visible light absorption capacity; as a result, the obtained membrane with self-cleaning ability showed the highest water flux recovery ratio (96.65%).

**Figure 12:**
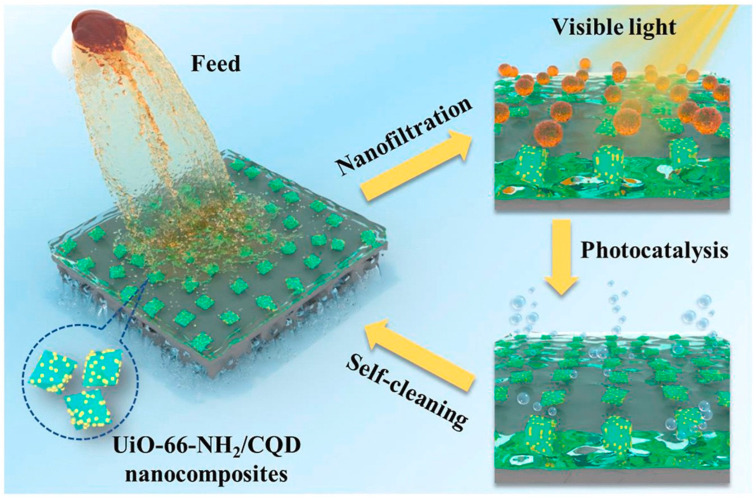
Photocatalytic TFN membrane with self-cleaning ability with the incorporation of UiO-66-NH_2_/CQDs [110].

Moreover, CDs can be composited with other nanocatalysts to enhance their photocatalytic property by improving visible light adsorption ability and suppressing the recombination of photogenerated electrons and holes [110,111]. A metal–organic framework (UiO-66-NH_2_) was composited with CQDs forming heterojunctions (UiO-66-NH_2_/CQDs) and were incorporated into TFN NF membranes by Zhao et al. [110]. The water permeance remarkably increased by 89.7% and efficiently rejected dyes. In addition, the hydrophilic CQDs increase the photocatalytic performance of UiO-66-NH_2_ by extending the wavelength range of absorbed visible light. The contaminants adsorbed on the membrane surface can be fast photocatalytic degraded in 10 min, even after 10 cycles of fouling, the water flux can be recovered to 92–99% of its initial state. All results demonstrated that the prepared membrane possessed excellent self-cleaning ability (Figure 12). Our previous work also reported self-cleaning NF membranes by incorporation of a CQD/TiO_2_ nanocomposite [112]. To ensure the stability of the TFN membrane, a PDA coating was pre-coated on the polysulfone substrate. As nanofillers, CQD/TiO_2_ nanocomposite increased membrane hydrophilicity, resulting in a high water permeance (11.2 L m^−2^ h^−1^ bar^−1^) and dye rejections (>99.1%). Moreover, CQDs enhance the visible light irradiated photocatalytic ability of CQD/TiO_2_ nanocomposite; consequently, the TFN membrane could nearly degrade the methyl blue adsorbed on the membrane surface after fouling. The flux recovery ratio was maintained at around 97–98% in the cyclic fouling-irradiation process, much higher than that of physical cleaning by pure water. This membrane achieved good stability, and the rejection of methyl blue was well preserved after photocatalytic degradation. The good dye desalination, self-cleaning performance, and stability of the prepared membranes made it suitable for dye wastewater treatment.

**Figure 13:**
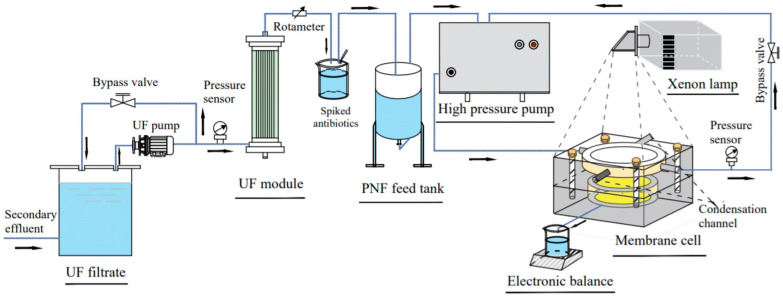
Schematic flow diagram of nanofiltration with the in situ light irradiation in a continuous dynamic process [114].

In most studies, the photocatalytic self-cleaning functions are realized mainly through ex situ self-cleaning, and the membranes are removed from the filtration cell for light exposure prior to re-insertion for flux recovery measurement. The inability to deliver light into the membrane operating module prevents the photocatalytic membranes from industrial applications. Therefore, Song et al. [113,114] designed a series of photocatalytic NF membranes based on NCQDs and g-C_3_N_4_, which simultaneously degrade and separate antibiotics in a continuous dynamic process (Figure 13). Take, for example, the membranes (named PNF-4) that owned a polyamide separation layer with the incorporation of NCQDs, and its outer surface was a mesoporous photocatalytic degradation layer (NCQDs modified g-C_3_N_4_, HNCCN-4) via assisted-vacuum dip-coating filtration [113]. The results showed that NCQDs inhibited the recombination of photoinduced electrons and holes and enlarged the adsorption regions of visible light, both boosting the photocatalytic activity of HNCCN-4. The antibiotics can be first degraded by the HNCCN-4 layer and further removed by the underlying polyamide layer during continuous dynamic filtration. As a result, the concentrations of trimethoprim and sulfamethoxazole were reduced to below two orders of magnitude in real water by the PNF-4. Meanwhile, the mesoporous photocatalytic layer had a negligible contribution to membrane resistance, and PNF-4 also showed a high water permeance of 23.64 L m^−2^ h^−1^ bar^−1^.

**Figure 14:**
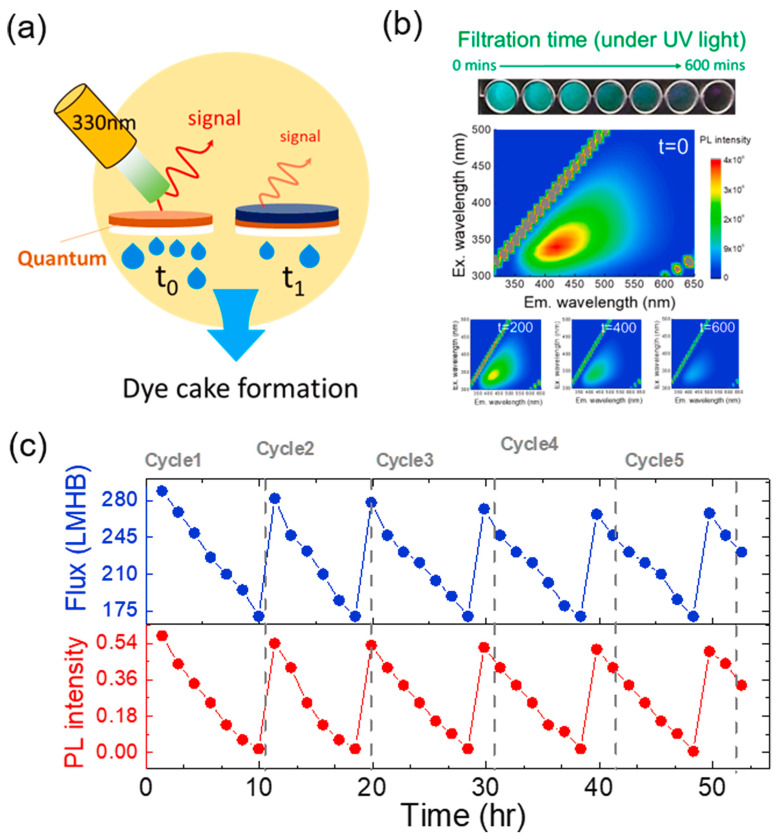
(**a**) Illustration of dye cake formation by PL. (**b**) PL maps of NGQD membranes during filtration. (**c**) Repeatability test of NGQD membranes using PL measurement [115].

In addition, the superior PL properties provide a facile method for in situ monitoring membrane fouling during the membrane separation process. Yeh et al. [115] utilized NCQDs to tune the microstructures of polyamide membranes and found that the stable PL emission character of NCQDs could be used to monitor dye fouling (Figure 14). Except for the remarkable water permeance of 289 L m^−2^ h^−1^ bar^−1^ and 99.96% rejection to methylene blue, the dye cake formation process on membranes could be tracked and recorded according to the fluorescence signals of NGQDs. The formation of dye cake resulted in gradually decreased PL intensity with the extension of filtration time. The positive correlation between filtration time and PL intensity indicates that NGQDs are sensitive probes for the detection of membrane fouling.

GQDs have been demonstrated to be cytotoxic under light irradiation, attributed to light-induced generation of reactive oxygen species (ROS). Photoexcited GQDs induced the electron–hole pair, which is responsible for the formation of ROS [116,117]. It is known that polydopamine (PDA) is obtained by oxidation of dopamine (DA). Due to the weak oxidation of oxygen, the polymerization of DA in air usually takes a long time, and the addition of oxidants can accelerate this polymerization process [118,119]. ROS are more active than molecular oxygen; therefore, benefiting from the ROS-generating activities of CQDs, Mi et al. [120] proposed a facile strategy to promote the fast formation of PDA by adopting NCQDs as a trigger. The addition of CQDs shortened the deposition time and was beneficial for the formation of a smooth and intact PDA coating layer. Under the optimized fabrication conditions, the NF membrane exhibited superior dye rejection of methyl blue (99.7%) and water permeance of 4.06 L m^−2^ h^−1^ bar^−1^. Moreover, the selective separation factor of methyl blue/NaCl was increased to 199.8, indicating good dye desalination ability. Similarly, Das et al. [121] adopted CDs to initiate the polymerization of norepinephrine and in situ synthesize MXene/poly(norepinephrine)/copper hybrid nanocomposites under UV light. The membrane was obtained via vacuum filtration and demonstrated excellent reduction of 4-nitrophenol.

In addition, there is attention to the role of CDs’ conductivity in enhancing membrane performance. Guo et al. [122] reported a conductive and loose TFN NF membrane by the synergistic manipulation of NGQDs, DA, and PIP via interfacial polymerization. The incorporation of N-GQDs simultaneously increased the membrane’s conductivity, hydrophilicity, and charge properties. Under an electric field, the charged and hydrophilic groups on NGQDs tended to migrate to the membrane pore surfaces, causing an instantaneous increase in membrane pore size and hydrophilicity. Moreover, the electrostatic repulsion between anions and the negative membrane surface was strengthened due to the enrichment of charged groups under an electric field. Therefore, when filtrating high salinity solution containing 15 g L^−1^ Na_2_SO_4_ and 127 g L^−1^ NaCl, the TFN-NF membrane achieved improvement in water permeance (17.30 L m^−2^ h^−1^ bar^−1^) and NaCl/Na_2_SO_4_ selectivity of 259.33 synchronously under an electric field.

## 5. Conclusions and Perspectives

In this review, we summarized the recent development of CDs-based nanocomposite membranes. The ultra-small size and good dispersibility made them compatible with polymer matrices; as a result, CDs are regarded as ideal nanofillers for fabricating nanocomposite membranes.

- CDs could regulate the structures of the resultant membranes. The hydrophilicity, reactivity, and charge properties of CDs, which are adjusted by their functional groups, would impact the microstructures, surface charge, and hydrophilicity of nanocomposite membranes.
- CDs endow the nanocomposite membranes with extra features. The optical and electric properties of CDs can be customizable to endow the nanocomposite membranes with self-cleaning and conductive performance.
- The incorporation of CDs can significantly enhance the resultant nanocomposite membranes’ performance, including permeation, rejection, selective separation, antifouling ability (or self-cleaning), and chlorine resistance. These improvements suitably position CDs-based nanocomposite membranes for various applications in NF, RO, UF, PV, membrane distillation, and ion exchange processes.

Despite many achievements that have been realized, some challenges still need to be addressed in the next stage: (1) Many methods such as pyrolysis, laser irradiation, electrochemical etching, microwave-assisted synthesis, and hydrothermal routes have been applied to synthesize CDs. However, the low yields of CDs limit their use in membrane fabrications, particularly in industry applications. It is essential to explore a simple, green, and large-scale method for synthesizing CDs. (2) The addition of CDs significantly changes the microstructure of separation layers in TFN membranes. However, even when using the same CDs and amine monomers, some form thin and smooth surfaces, while others form thick and rough surfaces. One of the possible reasons is the use of different naming conventions for CDs. Even though the name is the same, the properties of CDs might be different. In addition, more in-depth research about membrane formation mechanisms after the incorporation of CDs is required. (3) At present, CDs-based nanocomposite membranes are still dominated by water treatment membranes. The potential of CDs in other separation membranes should be fully explored. (4) CDs possess diverse sizes, functional groups, and compositions. Thus, the leakage and toxicity of these functionalized CDs during the membrane separation process should be thoroughly assessed.

In summary, these challenges would be overcome with more fundamental research, and we can expect that CDs-based nanocomposite membranes exhibit huge potential in the revolutionization of membrane separation.

## Figures and Tables

**Table 1:** Characteristics of CDs.

	Size (nm)	Crystallinity	Observed by TEM	Hydrophilicity	Properties
GQD [24,25]	lateral dimension < 20 nm with less than 10 graphene layers	highly crystalline	√	amphiphilic	fluorescence properties originate from quantum confinement and edge effect
CQD [24,25]	<10 nm	crystal lattices and chemical groups on the surface	√	hydrophilic or hydrophobic	photoluminescence and electronic bandgap depend on structural defects, heteroatom doping, vacancy defects, and quantum confinement effect
CND [26]	<10 nm	amorphous	√	highly hydrophilic	lack quantum confinement; photoluminescence behavior is related to the surface defects; strong electron-donating and accepting abilities
CPDs [22,27]	-	low	×	highly hydrophilic	abundant functional groups; polydispersity in structures; highly crosslinked network structure; photoluminescence originates from the surface state, subdomain state, molecular state, and crosslink-enhanced emission effect

**Table 2:** CDs-based TFN membranes prepared via interfacial polymerization.

Amine Monomer	CDs	Characters of Separation Layer Compared with Pristine One				Separation Performance and Application	Ref.
		Roughness	Thickness	Hydrophilicity	Surface Charge		
PIP	AGQDs	smoother	thinner	improved	decreased	J = 18.6 L m^−2^ h^−1^ bar^−1^ (increased by 60.9%) R_Na2SO4_ = 95.7% desalination	[43]
	GQDs	smoother	thinner	improved	decreased	J = 40.3 L m^−2^ h^−1^ bar^−1^; R_Na2SO4_ = 99.7%; selectivity of Cl^−^/SO_4_^2−^ = 309.5 desalination	[44]
	NGQDs	smoother	thinner	slightly decreased	slightly decreased	J = 12.4 L m^−2^ h^−1^ bar^−1^ R_CR_ = 99.5%; R_NaCl_ = 14.3% dye desalination	[45]
	GQDs	smoother	-	improved	decreased	J = 51.0 L m^−2^ h^−1^ bar^−1^ (increased by 580%) R_GR_ = 96.0%; R_OG_ = 80% dye removal	[37]
	AA-fGQDs	smoother	thinner	improved	depends on amino acid type	J = 27.3 L m^−2^ h^−1^ bar^−1^; R_Na2SO4_ = 97.9% (TFN-Asp-GQDs); highest antifouling ability (TFN-Cys-GQDs) desalination	[46]
	CNQDs	smoother	thinner	improved	increased	J = 13.7 L m^−2^ h^−1^ bar^−1^; RMB > 99%; dye removal	[47]
	PEI-CDs	smoother	thinner	increased	more positive	J = 15.3 L m^−2^ h^−1^ bar^−1^; R_MgSO4_ = 98.3% J = 30.9 L m^−2^ h^−1^ bar^−1^; R_Na2SO4_ = 99.4% desalination	[48]
	PS-CDs	unchanged	rougher		more negative		
	Na^+^-CQDs	rougher	thicker	improved	increased	J = 10.4 L m^−2^ h^−1^ bar^−1^; ${RSeO}_{3}^{2-}$ = 97.5%; ${RHAsO}_{4}^{2-}$ = 99.5%; ions removal	[49]
	NCQDs	smoother	-	improved	decreased	TFN-SCQDs achieved best water permeance J = 7.0 L m^−2^ h^−1^ bar^−1^; R_Na2SO4_ = 96.3%; TFN-NCQD had better rejection of divalent cations	[40]
	CCQDs	rougher			increased		
	SCQDs	rougher			increased		
	CDs-ZPEI_10k_	rougher	thicker	improved	unchanged	J = 11.4 L m^−2^ h^−1^ bar^−1^ (increased by 138%); R_Na2SO4_ = 98.1%; desalination	[50]
PIP + BP	GQDs	smoother	thinner	improved	increased	J = 59.6 L m^−2^ h^−1^ bar^−1^; R_direct black_ = 99.9%; R_NaCl_ = 2.6%; dye desalination	[51]
MPD	aGQDs	smoother	thinner	-	-	J = 3.80 L m^−2^ h^−1^ bar^−1^ (ethanol, increased by 44%) R_RDB_ = 99.0%; solvent resistance NF	[52]
	af-GQDs	smoother	thinner	-	-	J = 9.76 L m^−2^ h^−1^ bar^−1^ (ethanol); R_RDB_ = 98.0%; OSN	[53]
	GQDs	smoother	thinner	-	-	J = 22.6 L m^−2^ h^−1^ bar^−1^ (ethanol); R_RDB_ = 98.0%; solvent resistance NF	[54]
	af-GQDs	smoother	thinner			J = 13.5 L m^−2^ h^−1^ bar^−1^ (methanol); J = 6.98 L m^−2^ h^−1^ bar^−1^ (ethanol); J = 11.2 L m^−2^ h^−1^ bar^−1^ (dimethyl formamide); R_RDB_ = 99.2%; OSN	[55]
	GQDs	smoother	ultrathin	improved	increased	J = 32.1 L m^−2^ h^−1^ bar^−1^; R_Na2SO4_ = 99.6%; selectivity of Cl^−^/SO_4_^2−^ = 205.8; desalination	[56]
	NH_2_-GOQDs	smoother	-	improved	unchanged	J = 13.3 L m^−2^ h^−1^ bar^−1^, reverse salt flux = 6.0 g^−2^ h^−1^ (2 bar); FO	[57]
	N-GOQD	smoother	unchanged	improved	-	J = 1.7 L m^−2^ h^−1^ bar^−1^; R_NaCl_ = ~93%; RO	[58]
	GQDs	smoother	-	improved	-	J = 30.9 L m^−2^ h^−1^, reverse solute flux = 0.12 g L^−1^; FO	[59]
	CQDs-EA	rougher	thicker	improved	-	J = 5.50 L m^−2^ h^−1^ bar^−1^ (increased by 42.1%); R_NaCl_ = 98.0%; brackish water desalination	[60]
	GQD-_NH2_	rougher	thinner	improved	-	J = 11.1 L m^−2^ h^−1^ bar^−1^ (methanol); J = 5.9 L m^−2^ h^−1^ bar^−1^ (dimethyl formamide); OSN	[61]
	Na^+^-CQDs	rougher	thinner	-	-	J = 3.84 L m^−2^ h^−1^ bar^−1^; R_NaCl_ = 98.6%; J = 1.13 L m^−2^ h^−1^ bar^−1^; R_NaCl_ = 96.7%; brackish water desalination	[41]
	CQDs	smoother	thinner				
	BWD-NCQDs	rougher	thinner	improved	-	J = 8.2 L m^−2^ h^−1^ bar^−1^ (increased by 54.7%); reverse solute flux decreased to 3.81 g^−2^ h^−1^ (3 bar) FO	[62]
	GOQD	rougher	thinner	improved	-	J = 2.1 L m^−2^ h^−1^ bar^−1^; R_NaCl_ = 98.8%; RO	[63]
	N/S-GOQD	rougher	thicker	improved	increased	J = 5.9 L m^−2^ h^−1^ bar^−1^; R_NaCl_ = 97.1%; RO	[64]
	CDs	rougher	thinner	improved	-	J = 5.7 L m^−2^ h^−1^ bar^−1^; R_NaCl_ = 99.0%; RO	[65]
β-CD	GQDs	smoother	slightly thicker	improved	decreased	J = 474.7 L m^−2^ h^−1^ bar^−1^ (increased by 290%) R_EBT_ > 93.0%; R_CR_ > 93.0%; dye removal	[66]
	NGQDs	unchanged	thicker	-	-	CO_2_ permeance 174.5 GPU; CO_2_/N_2_ selectivity = 23.3 gas separation	[67]
PEI	QCQDs	smoother	thinner	improved	increased	J = 23.8 L m^−2^ h^−1^ bar^−1^; R_MgCl2_ = 95.6%; R_RB_ = 98.9%; R_trimethoprim_ = 99.7%; cationic small-sized contaminants removal	[68]
	GQDs-NH_2_	smoother	thinner	improved	increased	J = 11.9 L m^−2^ h^−1^ bar^−1^; separation factor of Mg^2+^/Li^+^ = 0.0359	[39]
TA	GQD	smoother	thinner	improved	increased	J = 11.6 L m^−2^ h^−1^ bar^−1^; R_CR_ = 99.8%; R_MB_ = 97.6%; R_NaCl_ = 17.2%; dye desalination	[69]
	NGQD	rougher	-	improved	-	n-butanol in feed (90%): total flux = 1000 g m^−2^ h^−1^ at 25 °C, water concentration in permeate 97.1 wt% PV	[70]
EDA + PVA	N-CDs	rougher	thinner	improved	-	total permeation flux of 3.15 kg m^−2^ h^−1^; ethanol/water separation factor of 1127 PV	[71]
